# Combined effect of ultrasound and vacuum impregnation for the modification of apple tissue enriched with aloe vera juice

**DOI:** 10.1016/j.ultsonch.2024.106812

**Published:** 2024-02-17

**Authors:** Magdalena Trusinska, Katarzyna Rybak, Federico Drudi, Urszula Tylewicz, Malgorzata Nowacka

**Affiliations:** a Department of Food Engineering and Process Management, Institute of Food Sciences, Warsaw University of Life Sciences- SGGW, Nowoursynowska 159c, Warsaw 02-776, Poland; b Department of Agricultural and Food Sciences, University of Bologna, Piazza Goidanich 60, Cesena 47521, Italy; c Interdepartmental Centre for Agri-Food Industrial Research, University of Bologna, Via Quinto Bucci 336, Cesena 47521, Italy

**Keywords:** Ultrasound treatment, Vacuum impregnation, Colour, Structure, Texture, Bioactive compounds

## Abstract

The aim of the work was to investigate how ultrasonic (US) treatment impacts on the physical and chemical properties of vacuum-impregnated apples. Apple slices were subjected to vacuum impregnation (VI) in an Aloe vera juice solution without additional treatments, serving as the reference material. Alternatively, ultrasound (US) treatments, at frequencies of 25 or 45 kHz, and durations of 10, 20, or 30 min, were employed as a pre-treatments before the VI process. The use of US processing enabled a significant increase in the efficiency of VI, without influencing in a significant way the color of the VI samples. The VI process led to a reduction in the content of bioactive compounds, in particular vitamin C and TPC decreased by 34 and 32 %, respectively. The use of US as a pre-treatment, in particular at 45 kHz for 20 or 30 min, led to a better preservation of these compounds (unchanged values for vitamin C and decrease by 23–26 % for TPC in comparison to the fresh samples). Through cluster analysis encompassing all assessed properties, it was evident that US treatment was beneficial for the processing, however the application of appropriate parameters of US treatment (frequency and time) had an impact on achieving similar quality to VI samples. The ultrasound treatment before vacuum impregnation may be suitable, however, the specific processing parameters should be defined for the obtained high quality of the final product.

## Highlights

• The vacuum impregnation (VI) process in aloe vera juice was conducted.
• The ultrasound treatment (US) was applied before VI to increase efficiency.
• Effect of US and VI process on physical and bioactive compounds were studied.
• Type of US processing (45 kHz, 20–30 min) has a positive impact on the quality of VI products.

## Graphical abstract

**Figure:**
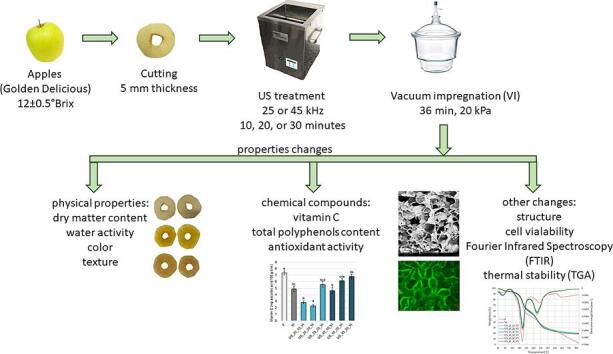

## 1 Introduction

The food industry has recently focused on designing functional, nutrient-rich food products driven by the growing demand for foods with health-promoting properties and increasing consumer awareness of the impact of food on human health. Currently, food should not only be consumed to satisfy hunger and provide essential elements, but also to enhance nutritional value, prevent nutrition-related diseases, and improve both physical and mental well-being. To meet these challenges novel mild and efficient processing techniques need to be investigated [1], [2], [3]. One of methods used to enrich food with desirable compounds is vacuum impregnation. It can be used to introduce compound such as: minerals, vitamins, probiotics, etc. into a porous tissue [2]. Vacuum impregnation process is based on filling the pores in the food tissue with a solution containing desired substances. It consists of two steps: first, the native gases and liquids are removed from the pores under vacuum and the pores expand as a result of the pressure gradient, and second, the impregnating solution flows into the pores due to the restoration of atmospheric pressure and opposite pressure gradient [4]. This mechanism is based on two phenomena: the hydrodynamic mechanism (HDM) and the deformation–relaxation phenomena (DRP) [5], [6].

Vacuum impregnation (VI) can be used to achieve not only nutritional, but also qualitative and technological functions [1]. For example, the VI process can improve the texture of food when used as a pre-treatment prior to drying, freezing, or frying [7]. It has been reported that the introduction of stabilizers such as pectin [8], [9] or zinc and calcium salts [9] into plant tissue before freezing has a positive effect on the quality of frozen-thawed products. Additionally, the combination of vacuum impregnation with osmotic dehydration (called vacuum osmotic dehydration) can led to a reduction in water activity and consequently to an improvement in the quality of further dried [10] or frozen product [9], [11]. Thanks to enhanced efficiency of heat conduction, vacuum impregnation pre-treatment may shorten the duration of thermal treatment such as pasteurization and sterilization, resulting in higher quality of the final product [2]. Another application of vacuum impregnation is to increase food stability by lowering pH. An acidic solution fills the pores and causes an increase in the diffusion rate of hydrogen ions, which lowers the pH of the treated products [3]. Due to oxygen removal from the pores, this method can be also applied to prevent color changes caused by enzymatic and oxidative browning [12]. Finally, the use of specific impregnation solution can contribute to changes in sensory properties, like aroma or taste [13].

Depending on the type of the treated material and the purpose of the vacuum impregnation, its efficiency can be improved either by optimising the process parameters [14], or by combining vacuum impregnation with other innovative food processing techniques which can be used as a pre-treatment, as a treatment carried out simultaneously with the process or as a post-treatment. Achieving a synergistic effect is the subject of numerous studies. The combined methods are tailored to the specific objectives, e.g. increase in mass transfer rate, extension of products shelf-life or increase in overall process efficiency [3]. One promising technique when combined with vacuum impregnation is ultrasound treatment (US).

Ultrasonic techniques have a wide range of applications in the food industry – they are used to support processes such as extraction, drying, filtration, freezing, thawing, emulsifying, cutting, foaming, homogenisation or cooking. The main objectives that can be achieved by using ultrasound in food technology are to reduce processing time, save energy and extend shelf-life [15], [16]. Ultrasonic techniques with a power above 1 W/cm^2^ are used as a method for food preservation. They are particularly useful in the production of minimally processed foods. High-power, low-frequency ultrasound (20–100 kHz) produces a cavitation effect [17].

Cavitation is a phenomenon caused by a changing pressure field in a liquid. The essence of cavitation is the formation, growth and disappearance of bubbles containing the vapour of a given liquid, gas or vapour-gas mixture. At low pressure the volume of the bubbles increases, while at high pressure it decreases. Ultrasound causes acoustic cavitation with the formation of vacuum bubbles. The implosion of the bubbles causes high frictional stresses. This cause the disintegration of cell structures and, in particular, perforation of cell walls and membranes, which can lead to the inactivation of microorganisms. Perforation is not only useful for food sanitization, but also facilitates the migration of water and other components from the tissue, enabling shorter heat treatment times, and thus reducing costs and nutrient losses [15]. One of the main applications of ultrasound treatment in food technology is the acceleration of mass transfer processes. Many published papers have shown that ultrasound can increase the efficiency of processes such as drying, extraction, freezing or osmotic dehydration [18]. Due to the mass transfer acceleration mechanisms, ultrasound treatment appears to be a promising technique also for supporting vacuum impregnation processes, both before and during the process [19].

Aloe vera, a member of the Liliaceae family, is a perennial plant with a cactus-like fleshy leaves. Aloe vera, in particular aloe vera gel consists mostly of water (98.5 % – 99.5 %), the remaining solid part contains more than 200 different components, mostly polysaccharides, and other minor compound such as soluble sugars, glycoproteins, phenolic anthraquinones, flavonoids, flavonols, enzymes, minerals, amino acids, sterols, saponins, and vitamins [20]. Aloe vera has many health promoting properties such as lowering cholesterol and triglyceride levels in human blood, anti-inflammatory and anti-biotic properties, beneficial effects against gastrointestinal, renal and cardiovascular diseases [21], [22]. This positive effect could probably be attributed to the presence of the bioactive components, in particular polysaccharides and phenolic compounds [23].

Recently, Aloe vera gel has been used as an impregnating solution, showing good potential in enhancing the nutritional properties of apple slices [24], however little is known on the effect of Aloe vera gel or juice impregnation on other chemical and physicochemical properties of food matrix.

Therefore, the aim of this study was to determine the impact of ultrasonic treatments with different parameters on selected physicochemical properties of apples subjected to vacuum impregnation in Aloe vera juice. To evaluate the process and changes in plant tissue different analysis such as dry matter content, water activity, color, texture, structure, cell viability, chemical compounds as vitamin C, total polyphenols content, antioxidant activity, Fourier Infrared Spectroscopy (FTIR) as well as thermal stability and decomposition characteristics were performed.

## 2 Material and methods

### 2.1 Material

The material used for the experiment was certified organic Golden Delicious apples (with total soluble solid-TSS content of 12±0.5 Brix), acquired in a single purchase from a local store in Cesena, Italy. Prior to the trial, the fruits were cold stored at a temperature of 4±1 °C, maintaining a relative humidity of 80 %. After removal from the storage the raw material underwent a thorough washing process using tap water and were subsequently cut into 5 mm thick slices. The removal of seeds and peel was accomplished using a corer and a sharp knife, respectively. Ultrasound treatment and the impregnation process were carried out in aloe vera juice acquired from Aloe Barbadensis Miller leaves from controlled organic farming – Alba Aloe Vera Organic 99.9 % Bio − 1°Brix (Benessence by Natura Srl, Termoli, Italy). To prevent the phenomenon of osmosis during vacuum impregnation, an isotonic solution was prepared with the same osmotic potential as the sample subjected to the process. The solution was prepared by dissolving 12 g of trehalose (from EXACTA + OPTECH Labcenter S.p.A., Italy) in 1 % aloe vera juice, resulting in 13 % concentration of the final solution with 13°Brix. Trehalose was used, instead of sucrose, because of its proved protective effect on cell membranes during the abiotic stress, allowing tissue viability and structure preservation [25].

### 2.2 Technological process

#### 2.2.1 Ultrasound treatment (US)

Ultrasound treatment was performed using the immersion method. 100 g of material was weighed with an accuracy of ±0.01 g and placed in glass beakers, carefully covered with a plastic net to ensure complete immersion of the samples in the solution during sonication. The solution prepared for vacuum impregnation was then added so that the mass ratio of raw material to solution was 1:7. The beakers were placed in an ultrasonic bath model Elmasonic Xtra ST 600H (Elma Schmidbauer GmbH, Singen, Germany). The treatment was carried out for 10, 20, or 30 min at an ultrasonic frequency of 25 or 45 kHz and a power of 800 or 900 W, respectively. During the process the temperature was kept constant. After processing, the beaker was removed from the water, the outside of the beaker was dried and placed in an impregnation chamber.

#### 2.2.2 Vacuum impregnation process (VI)

The vacuum impregnation (VI) process was employed after sonication treatment, resulting in the acquisition of six different samples at the end of processing (except for the reference material) (Table 1).

**Table 1:** Abbreviations for samples subjected to ultrasound treatment and vacuum impregnation process.

Abbreviations	VI	US frequency [kHz]	US Time [min]
F	–	–	–
VI	+	–	–
US25_10VI	+	25	10
US25_20VI	+	25	20
US25_30VI	+	25	30
US45_10VI	+	45	10
US45_20VI	+	45	20
US45_30VI	+	45	30

The VI process was performed in a sealed chamber connected to a vacuum pump and an automatic vacuum control system (AVCS, S.I.A., Bologna, Italy), all maintained at a constant temperature of 25±1 °C. Apple slices, weighed and placed on a metal frame to avoid contact between samples, were positioned in 800 cm^3^ beakers. These samples were then treatment with a solution, maintaining a solution-to-mass ratio of 1:7 (v/m). The VI treatment lasted 36 min, with a gradual increase in vacuum over 8 min (100–80 kPa for 2 min, 80–60 kPa for 2 min, 60–40 kPa for 2 min, 40–20 kPa for 2 min). The samples were then kept under a pressure of 20 kPa (absolute pressure) for 10 min, followed by a gradual restoration of atmospheric pressure over 8 min. The vacuum impregnation was followed by a relaxation phase of 10 min. After the treatment, the apple slices were carefully removed from the solution, gently dried on filter paper, and weighted. Six replicates were performed for each sample. The weight gain (*WG*) during the process was calculated using the formula according to Tappi et al. [26]:

(1) $$\mathrm{WG}=100∙\frac{m-m_{0}}{m_{0}}$$

where:

*m* – the mass of the sample after vacuum treatment [g].

*m_0_* – the initial mass of the sample before vacuum treatment [g].

### 2.3 Main properties determination

#### 2.3.1 Dry matter content

A vacuum dryer with low pressure (≤100 mmHg) was used for the gravimetric determination of dry matter content in fresh and treated fruits. The drying process was carried out at a temperature of 70 °C for a duration of 24 h [27]. This measurement was performed in triplicate.

#### 2.3.2 Water activity

The water activity of the samples was measured using an AquaLab Series3TE dew point hygrometer (Decagon Devices, Inc., Pullman, Washington, USA) with a precision level of ±0.001. This measurement was performed in duplicate for each sample, maintaining a temperature of 25±1 °C.

#### 2.3.3 Colour

The color was evaluated with a Colorflex colorimeter (Hunterlab, USA), working in the CIE Lab* mode. In this system, L* stands for brightness, a* for the color chromaticity coordinate from green to red, and b* for the color chromaticity coordinate from blue to yellow. Prior to measurement, the colorimeter was calibrated using black and white standards. The measurement consisted of ten repetitions for each material, using a D65 medium daylight source and a standard 10° observer. Based on the data obtained, the total color differences (ΔE) and the browning index (BI) were calculated for each sample, with reference to the color of fresh apple tissue [28].

#### 2.3.4 Texture

The texture of apple was analyzed using a Texture Analyser TA HDi500 (Stable Micro Systems, Surrey, UK). Textural characteristics were assessed through a compression test, wherein apple samples, positioned on the platform, were compressed at various points using an aluminum cylinder probe with a 5 mm diameter, moving at a head speed of 1 mm/s. The procedure concluded upon reaching 50 % of the sample height. Hardness was expressed as the maximum force required to compress the apple samples [29]. Twenty repetitions were carried out for each sample.

#### 2.3.5 Structure

The investigation of internal structural changes in the apple samples was performed using a Phenom XL scanning electron microscope (Phenom World, Eindhoven, the Netherlands). To facilitate observation, the material was subjected to freeze-drying process to eliminate water from the tissues. Slices were placed in a freezer at –40 °C for 48 h. Freeze-drying was carried out in a Lio2000 freeze dryer (Cinque Pascal S.r.l., Milan, Italy) with specific parameters: shelf temperature of 25 °C, pressure of 22 Pa, condenser temperature of –47 °C, and a duration of 48 h. Following the process, the material was sealed in light and air barrier packaging. For the analysis, small sections of freeze-dried plant tissue (0.5 x 0.5 cm), cut with a razor blade, were affixed to carbon tape and a metal table. To improve electrical conductivity, a 5 nm gold layer was applied to these samples through the 25 s sputter coating process using the Cressington 108 auto system (Cressington Scientific Instruments, Watford, UK). Microscopic observations were carried out at a voltage of 10 kV and a medium vacuum of 60 Pa. Each sample was documented with four cross-sectional photos at a magnification of 200x.

#### 2.3.6 Cell viability

Fluorescein diacetate was used to assess cell viability, in particular to determine whether living cells were present (indicated by green fluorescence). A stock solution of 10^−4^ M FDA was prepared in water and stored at 4 °C. Apple pieces, each 1 mm thick, were incubated for 5 min in the dark at 25 °C in a dye solution prepared with an isotonic concentration of sucrose. Following incubation, the samples were carefully rinsed with deionized water and examined under fluorescent light using a Nikon microscope (Eclipse TieU, Nikon Co, Japan) equipped with a Nikon digital video camera (digital sight DS-Qi1Mc, Nikon Co, Japan) with green filter with excitation at 495 nm and emission at 518 nm [30]. Each sample was documented with approximately 6 to 8 photos at a magnification of 10.

### 2.4 Chemical analysis

#### 2.4.1 Vitamin C content

The determination of vitamin C content was carried out using the UPLC-PDA system (WATERS Acquity H-Class, Milford, MA, USA), according to the method described by Spinola et al. [31]. In this procedure, 10 ml of a cold extraction solution (3 % metaphosphoric acid, 8 % acetic acid, 1 mM EDTA solution) was added to 0.05 g of the material and stirred for 10 min on a laboratory shaker. The solution was then centrifuged (6000 rpm, 4 °C, 5 min), and the supernatant was filtered through syringe filters (0.22 μm, CR-PTFE) before being diluted twice with eluent (Milli-Q water with 0.1 % formic acid). The determination employed a WATERS Acquity UPLC HSS T3 chromatographic column (2.1×100 mm, 1.8 μm; Waters, Ireland) with a mobile phase flow rate of 0.25 ml/min. The column and sample thermostat temperatures were maintained at 25 and 4 °C, respectively. The spectrum was analysed at a wavelength of 245 nm. The L-ascorbic acid content was calculated based on the calibration curve in the range from 0.025 to 5 mg/mL^1^ (r ^2^ ≤0.9997) for the L (-) ascorbic acid analytical standard. Each sample was analysed twice for accuracy.

#### 2.4.2 Total phenolic content (TPC)

##### 2.4.2.1 Preparation of the apple extract

In preparation for chemical analyses, the plant material was subjected to a freeze-drying process, as described in section 2.3.5, with the intent of standardizing samples and optimizing extractability. The dried apples were grounded using an A 11 basic analytical grinder (Ika-Werke, Staufen, Germany). Approximately 0.3 g of the powdered material was weighed into a 15 ml falcon, and 10 ml ethanol acidified with 0.1 N HCl (85:15) was added. Extraction took place over a 12-hour period at room temperature, with limited exposure to light, using a Multi Reax shaker (Heidolph Instruments, Germany). The solution was then centrifuged in a MegaStar 600 laboratory centrifuge (VWR, Leuven, Belgium, 4350 rpm/min, 2 min). This extraction procedure was repeated twice for each sample, ensuring thorough and replicable analysis.

##### 2.4.2.2 TPC analysis

The total phenolics content in apples was assessed by a spectrophotometric method, using a color reaction with the Folin-Ciocalteau reagent [32]. The extracts were twice diluted with distilled water, and reactions were conducted in 96-well plates. A 5-fold diluted Folin Ciocalteau reagent (40 µl) was added to 10 µl of the extract, followed by the addition of 250 µl of a 7 % sodium carbonate solution after 3 min. The solution was then incubated for 60 min at room temperature without exposure to light. Absorbance at 750 nm was measured using a Multiskan Sky plate reader (Thermo Electron Co., Waltham, MA, USA). The absorbance of the blank sample, in which the extract was replaced by the extraction reagent, was also measured. Two repetitions were performed for each tested extract. To quantify the polyphenol content, a calibration curve was prepared using chlorogenic acid (Sigma Aldrich, Switzerland) in the concentration range of 0–100 g/ml. Results were expressed as the amount of milligrams of chlorogenic acid per 100 g of dry matter.

#### 2.4.3 ABTS assay scavenging activity

The evaluation of the antioxidant activity in the tested samples focused on evaluating their capability to reduce the ABTS^•+^ cation radical. The initial solution was prepared by dissolving 38.4 mg of 2,2′-Azino-bis(3-ethylbenzthiazoline-6-sulfonic acid) and 6.6 mg of potassium persulfate in 10 ml of distilled water, which was then left at 4 °C for 12 h. The working solution, generated immediately before analysis, consisted of dilution of the starting solution with an 80 % ethanol solution. For the analysis, 10, 20, 30, and 40 µl of the sample extract were combined with 3 ml of the radical solution in reaction tubes. Following a 6-min interval, absorbance was measured at a wavelength of 734 nm using a Helios Thermo Electron 7.03 spectrophotometer (Thermo Electron Corporation, USA) [33]. Antioxidant activity was determined by measuring the reduction in absorbance of the radical solution in the presence of an antioxidant and expressed in milligrams of Trolox per gram of sample dry matter. The assay was performed in duplicate.

#### 2.4.4 Fourier Infrared Spectroscopy (FTIR)

Infrared spectra measurements were performed using a Cary 630 spectrometer (Agilent Technologies Inc., Santa Clara, CA, USA) equipped with a single reflection diamond interface (ATR – attenuated total reflectance). The dried material was placed in the measuring window and pressed against the crystal. The spectra were captured in the wavenumber range of 4000–650 cm^−1^, comprising 32 scans at a resolution of 4 cm^−1^ [34]. After each measurement, the background was set to zero and the crystal was cleaned with an isopropanol solution. Data analysis was carried out using MicroLab FTIR software, and each sample underwent three separate measurements.

#### 2.4.5 Thermogravimetric analysis (TGA)

The thermal stability and decomposition characteristics of the dried samples were analyzed using a TGA/DSC 3+ thermogravimeter (Mettler Toledo, Greifensee, Switzerland). Approximately 5 to 7 mg of the material was weighed into 70 µl aluminium oxide crucibles and subjected to heating from 30 to 600 °C at a rate of 5 °C/min in a nitrogen atmosphere (50 ml/min) [35]. The TG and DTG curves were derived from the differential values of the mass loss with respect to temperature. Thermograms (TGA/DTG) were assessed using the STAR software (version 16.10) from Mettler Evaluation. Each sample underwent two replicates for robust analysis.

### 2.5 Statistical analysis

Statistical analysis was carried out using STATISTICA 13 software (StatSoft Inc., Tulsa, Oklahoma, USA). The relationships between the various properties of apples and the applied processing were examined through one-way ANOVA analysis of variance. To identify statistically significant differences among samples, a multiple comparisons analysis employing Tukey's test was performed. The existence of correlations between the given properties was also verified. The significance level for statistical inference was set at α = 0.05. For a comprehensive evaluation of the results, considering the analysed physicochemical properties, both cluster analysis and principal components analysis were conducted. Ward's method was applied along with the use of the Euclidean distance. The outcomes of the cluster analysis were then visually depicted on the dendrogram.

## 3 Results and discussion

### 3.1 Efficiency of vacuum impregnation process after US treatment of apples

**Fig. 1:**
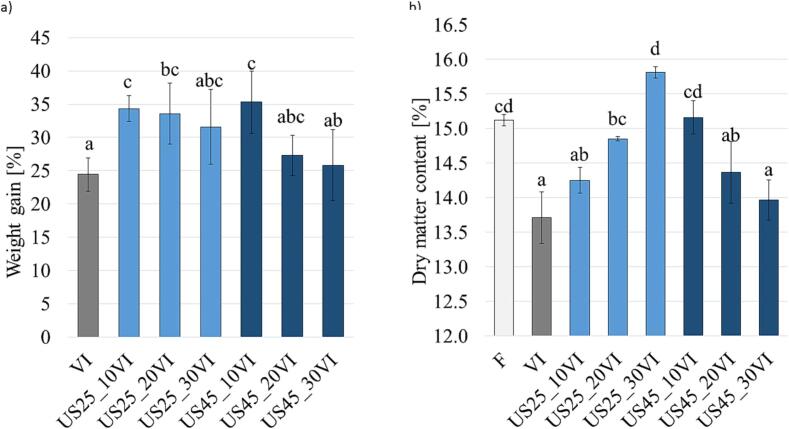
Effect of vacuum impregnation with the use of different treatment conditions of US treatment on a) mass gain; b) dry matter content, different letters above the columns indicate statistical differences (p < 0.05).

The vacuum impregnation technique enables the introduction of the impregnating solution into the pores or the intercellular spaces of the tissue [36]. The process efficiency was assessed based on mass gain through the VI. Fig. 1a shows the effect of different treatments on mass gain. The mass of vacuum impregnated apple tissue without the application of US pre-treatment increased by 24.41 % and the use of ultrasound pre-treatment at 25 or 45 kHz and a time of 10, 20 or 30 min resulted in weight gains that ranged from 25.82±5.35 % (45 kHz for 30 min) to 35.32±4.68 % (45 kHz for 10 min). The use of US for 20 or 30 min caused slightly higher mass gain at 25 kHz rather than 45 kHz. This may be due to the fact that at lower ultrasound frequencies, the cycles of compression and expansion in the tissue are longer, so the bubbles reach the size necessary to induce rapid cavitation. As a consequence, there is a greater rapture of the cells, which can accelerate the vacuum impregnation process as well as other mass exchange processes [37], [38].

Fig. 1b shows the dry matter content of the apple tissue. Apples are characterized by high porosity. The large intercellular spaces make these fruits susceptible to compression and expansion cycles during ultrasonic treatment, resulting in moisture loss [39]. As a result of ultrasonic treatment, the dry matter content increased significantly in three cases (25 kHz for 20 min; 25 kHz for 30 min; 45 kHz for 10 min) in comparison to samples subjected only to VI process. Subsequently, more impregnating solution can penetrate the pores, which explains the increase in dry matter content in some cases [40]. The use of ultrasound may induce unintended significant structural alterations, causing not only water loss but also a significant loss of substances dissolved in the cell juice, such as vitamins, sugars or organic acids. This phenomenon was demonstrated by Mierzwa et al. [19] when employing ultrasound during both the reduced pressure and relaxation stages of vacuum impregnation. The content of ascorbic acid in the product was significantly lower than when ultrasound was applied during one of the stages of vacuum impregnation. In turn, insufficient exposure time or low ultrasound frequency might lead to slight changes in tissue properties [41].

### 3.2 Physical properties of apples subjected to ultrasound treatment and vacuum impregnation process

The effects of the ultrasound treatment and vacuum impregnation process on apple tissue physical properties such as water activity, colour and texture are presented in Table 2.

**Table 2:** Water activity (a_w_), color parameters (L*, ΔE calculated in comparison to fresh apple tissue, BI – browning index) and texture (Max force [N]) of apple tissue subjected to the different treatment conditions of ultrasound treatment and vacuum impregnation process, different letters in column indicate statistical differences (p < 0.05).

Sample	a_w_	L*	ΔE	BI	Max force [N]
F	0.987±0.001 ^d^	78.63±2.22^b^	–	33.56±2.16 ^a^	10.80±1.83 ^a^
VI	0.983±0.001 ^bc^	50.35±6.55 ^a^	28.93±6.61 ^a^	46.02±7.14^b^	10.99±2.40 ^a^
US25_10VI	0.985±0.001 ^cd^	45.90±2.78 ^a^	33.06±2.96 ^a^	48.97±4.01^b^	10.06±2.72 ^a^
US25_20VI	0.982±0.001 ^bc^	47.03±1.92 ^a^	31.80±2.02 ^a^	49.90±4.10 ^bc^	12.23±3.34 ^a^
US25_30VI	0.982±0.001 ^bc^	47.38±4.64 ^a^	31.54±4.67 ^a^	51.68±5.01 ^bc^	11.83±2.83 ^a^
US45_10VI	0.984±0.001 ^bc^	46.04±3.66 ^a^	32.75±3.67 ^a^	57.20±8.39^c^	12.01±2.66 ^a^
US45_20VI	0.983±0.001 ^bc^	44.90±3.09 ^a^	34.17±3.33 ^a^	46.42±4.40^b^	11.22±2.35 ^a^
US45_30VI	0.979±0.001 ^a^	48.77±5.51 ^a^	30.21±5.37 ^a^	52.31±6.97 ^bc^	12.00±2.75 ^a^

The water activity of fresh apple was 0.987±0.001, while that of vacuum impregnated apple was significantly lower and equal to 0.983±0.001. In most cases, the vacuum impregnated samples with or without US treatment caused a statistically significant decrease in the value of this parameter compared to the fresh fruit (an exception was the US treatment of 25 kHz for 10 min). One of the mechanisms caused by ultrasound is sponge effect. Compression and expansion occurring alternately inside the material when ultrasonic is applied to the sample results in the formation of microscopic channels through which moisture can be removed more easily [16]. As a result of ultrasonic pre-treatment, moisture transport may be facilitated, which may lead to the higher removal of air and free water from the tissue during vacuum impregnation. Due to this mechanism, ultrasound has been used to support mass transfer processes. Kowalski et al. [42] and Kowalski et al. [43] subjected raspberries and apples to hot-air drying, respectively. In both cases, researchers showed a reduction in the water activity of dried fruit when the process was supported by ultrasound with a power of 100 or 200 W, respectively. However, the effect of ultrasound on porous tissue subjected to vacuum impregnation is not clear. In the US treated apples with frequency of 25 kHz for 10 min the water activity did not decrease significantly compared to the fresh material. A similar result was observed in the study by Mierzwa et al. [19], in which potato cubes previously subjected to vacuum impregnation were dried with or without the use of US. Water activity between fresh potatoes, potatoes subjected to VI and potatoes subjected to VI with US treatment did not differ significantly [19]. This indicates the complicated nature of the impact of ultrasound on the plant tissue and mostly depends on the applied treatment parameters.

**Fig. 2:**
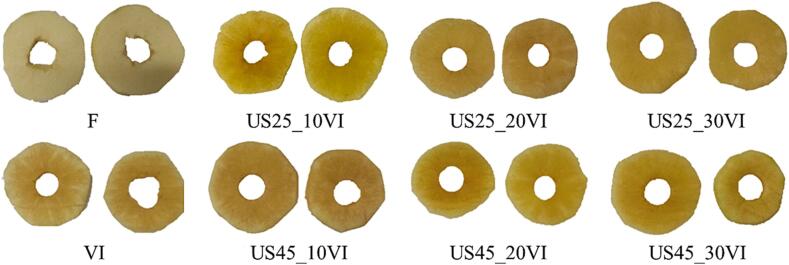
Macroscopic photographs of fresh and vacuum-impregnated apples subjected to the different treatment conditions of ultrasound treatment and vacuum impregnation process.

The color of food products is of great importance as it plays a crucial role in the evaluation of food quality and food acceptance by consumer. The color of the apple tissue after the VI process applied with or without US treatment changed, and in some cases the apple looked like a pineapple after impregnation (Fig. 2). Considering the results obtained for color parameters, it can be seen that the applied treatment resulted in the greatest changes for the parameter L*. The L* parameter value for fresh fruit was 78.63±2.22, which was significantly higher than the value for the material subjected to VI, which was equal to 50.35±6.55 (Table 2). The reduction in the L* parameter value observed in vacuum-impregnated apples could be due to the infusion of aloe vera juice solution into the apple's pores. Furtheremore, the US treatemnt applied before VI process resulted in higher color changes, resulting in lower L* parameter in the range of 44.90–48.77, which could be related to the cavitation and sponge effect [44]. The values of the L* parameter decreased by 36 % as a result of VI alone and by 37.9–42.9 % as a result of the additional US treatment applied before VI. However, no significant changes were observed between the samples subjected to VI process alone and with US treatment. Similar observations were made by Mashkour et al. [45], who found no significant differences in color between potatoes subjected to VI at a vacuum pressure of 30 kPa and potatoes subjected to the same treatment supported by US of different power (100, 200, 300 W), frequency (37, 80 kHz) and time (15–45 min). Furtheremore, a similar observation was done also for apples impregnated in a sucrose solution with ascorbic acid, green tea extract or a combination of these substances [46], or impregnated apples in a sucrose solution with ascorbic acid, calcium lactate or a combination of these two substances [26]. As a potential reason for the darkening of the samples due to the VI process, the literature indicated changes in the structural properties of the tissue that result from subjecting the tissue to a vacuum, and partially replacing the gas with a liquid, which promotes changes in the refractive index [26], [46], [47]. Nevertheless, it is worth noting that in our study, after the VI process, the standard deviation for the color parameters was relatively high, which indicates uneven saturation of the apple tissue with juice components (Fig. 2).

Changes in the total color difference (ΔE) parameter were also noticed, whose values ranged from 28.93 to 34.17. A value above 5 signifies a distinct contrast in color and observers perceive clearly different colors. The higher the value of this indicator, the greater the color difference between the compared materials [48]. Regardless of the US treatment and their parameters applied to VI, the total color difference consistently exceeded value of 5. This means that the consumer will have the impression of a completely different color of the product, which is also visible in the product photos (Fig. 2).

In order to further investigate the color changes occurring during vacuum impregnation with US treatment, the browning index -BI, which characterizes the intensity of the brown color, was calculated. This BI index is the result of the activity of enzymes found in the apple tissue, which have the potential to induce adverse alterations in both appearance and sensory attributes [49]. For fresh apples, the BI was registered at 33.56, while for VI samples the index increased significantly of about 37 % to the value of 46.02. In the cases with additional supportive US treatments, the BI reached higher values even of 57.20 (a rise of 70.4 %). This heightened intensity in brown coloration among these variations is visually noticeable in macroscopic images of the samples (Fig. 2). Moreover, a negative correlation (r = -0.86, p < 0.05) was found between BI and L* value, which means that an increase in L* value corresponds to a decrease in average BI values.

To assess the influence of the VI and US treatments on the hardness of apple tissue, the compression test was performed, showing the maximum force required to achieve a specified deformation (Table 2). The measured hardness for fresh and VI apples was similar and equal to 10.80±1.83 and 10.99±2.40 N, respectively. Analyzing the average values of texture parameter, it can be seen that the use of US in most cases slightly increased the hardness of the material, however these changes were not statistically significant. Yılmaz and Bilek [50] noticed, instead, a significant increase in the hardness of vacuum-impregnated apples as a result of ultrasonic treatment in calcium lactate and black carrot phenolics solution. In this case US treatment increase the Ca ions in the apple tissue and resulted in higher hardness of the apple discs.

### 3.3 Influence of ultrasound application and vacuum impregnation process on cell viability and apple structure

**Fig. 3:**
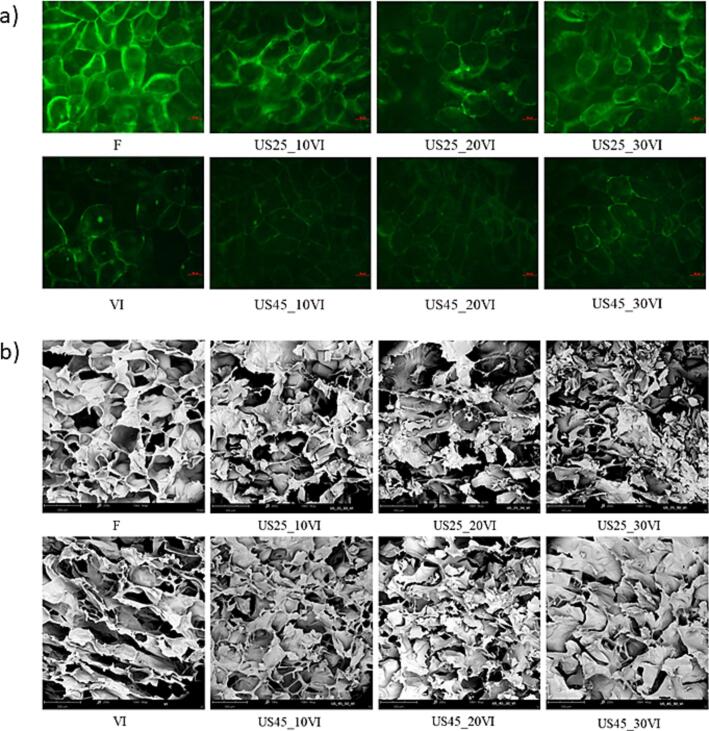
Microscopy images of apple tissue subjected to the different treatment conditions of ultrasound treatment and vacuum impregnation process a) followed by staining with FDA; b) made using SEM.

The images presented in Fig. 3 shows photographs of apple tissue treated with fluorescein diacetate (FDA) solution (Fig. 3a), used to evaluate the viability of apple tissue cells after VI and US treatment, and photographes obtained by Scanning Electron Microscope (SEM) (Fig. 3b) to evaluate the changes in the tissue structure. In the FDA solution, a living cell exhibits a distinctive light green fluorescence [26]. The cell viability may depend on the characteristics of the impregnating solution, especially factors like its pH or the concentration of dissolved substances within it [26], [51].

Vacuum impregnation of apple tissue in aloe vera juice caused partial damage to the cellular structure, but some cells retained their viability as light green stained viable cells could still be observed after treatment. The preservation of cell viability during vacuum impregnation was also demonstrated by other researchers [26], [52], [53]. When ultrasonic treatment was applied before vacuum impregnation, a partial loss of cell viability was also observed (Fig. 3a). A higher green color intensity indicates greater cell survival when using a frequency of 25 rather than 45 kHz. However, no clear effect of ultrasound treatment time on cell survival was observed in literature [54]. A different observation was made for whole or cut cranberry fruits, which was subjected to ultrasonic treatment at a frequency of 21 kHz for 30 or 60 min. Ultrasonic treatment for 30 min using whole or cut fruits did not significantly affect cell viability when 61.5 % sucrose or 30 % sucrose with 0.1 % steviol glycosides solutions were used. After a 60-min ultrasonic treatment, a significant loss of cell viability was observed, and when a sucrose solution without the addition of steviol glycosides was used, the loss of cell viability occurred to a greater extent, which was related to the use of a higher concentration of sucrose solution [55].

Accordingly, the changes in the structure of apple tissue subjected to VI and US treatment were also observed. Fresh apples showed a porous internal structure composed of numerous closely packed cells with small intercellular spaces. Vacuum impregnation led to cell deformation and a narrowing of the spaces between the pores, a phenomenon previously observed in other impregnated fruits like strawberries [56]. The alteration in cell shape and shrinkage can be attributed to the loss of native liquid from the tissue, resulting from the fracturing of cell walls after the impregnation process. Furthermore, the use of ultrasound before vacuum impregnation led to a loosening of the structure and the formation of microchannels inside the tissue. When a frequency of 45 kHz was used, more pores of smaller sizes were formed, while fewer pores of larger and irregular shape were formed with the ultrasonic treatment at a frequency of 25 kHz. The differences in structural changes when using different frequencies may be due to the fact that at a lower ultrasound frequency (25 kHz), the bubbles formed by cavitation become larger, resulting in large pores in the tissue [57]. Similar changes in apple tissue caused by ultrasound treatment were observed by Pieczywek et al. [58]. In the study, US treatment at a frequency of 20 kHz was used for 7.5, 15 or 30 min, and the longer the treatment lasted, the larger pores could be detected due to the disruption of cell walls [58]. This effect may be difficult to determine due to the empty space occupied by the impregnating solution. A higher frequency of ultrasonic treatment (45 kHz) results in the formation of more smaller bubbles as a result of cavitation. Treatment of the apple tissue with ultrasound before VI, regardless of the parameters used, resulted in the creation of a space inside the tissue into which the impregnating solution could penetrate. Similar changes in apple tissue as a result of sonication were also reported by Mieszczakowska-Frąć et al. [59] and Rajewska and Mierzwa [39].

### 3.4 Bioactive compounds of apples subjected to ultrasound treatment and vacuum impregnation process (vitamin C, total phenolic content, antioxidant activity)

**Fig. 4:**
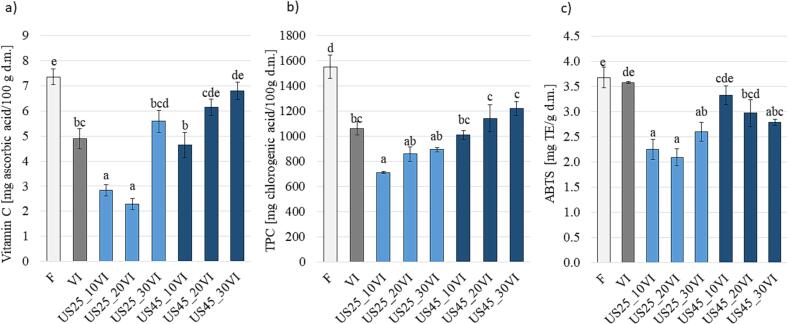
Chemical changes of a) vitamin C content; b) total phenolic content (TPC); c) antioxidant activity (with ABTS) of apple tissue subjected to the different treatment conditions of ultrasound treatment and vacuum impregnation process, different letters in column indicate statistical differences (p < 0.05).

Fig. 4 shows the changes in vitamin C (Fig. 4a), total phenolic content (TPC) (Fig. 4b) and in antioxidant activity (Fig. 4c) in apple tissue subjected to the different conditions of ultrasound treatment and vacuum impregnation process. Fresh apple samples presented the highest content of both vitamin C and TPC, respectively 7.4±0.3 mg/100 g d.m. and 1553±93 mg/100 g d.m. Vacuum impregnation process caused a reduction of vitamin C by 34 %, similarly TPC decreased by 32 %. The decrease of vitamin C and TPC in VI apples was probably due to lower concentration of these compounds in aloe vera juice used for impregnation in comparison to the apple tissue, causing their possible leakage into the impregnating solution. Regarding the vitamin C (Fig. 4a), the statistical analysis showed that content did not significantly decrease compared to fresh apple only when vacuum impregnation was preceded by ultrasonic treatment at a frequency of 45 kHz for 20 or 30 min. In all remaining variants, the vitamin C content was significantly lower than in fresh fruit. In general, vitamin C was better retained in the ultrasound impregnated apples with increasing frequency and duration of ultrasound exposure. However, significantly higher vitamin C content was obtained only in the impregnated apple pretreated with US at 45 kHz for 30 min compared to the vacuum impregnated apples without preliminary treatment. Similar results were obtained by Amanor-Atiemoh et al. [60] in apples osmodehydrated in 30 % glucose solution. Ultrasound assisted osmotic dehydration (20 kHz, 300 W/L) allowed a better vitamin C retention (46.1 %) compared to the osmotic dehydration process alone (25.6 %). Mierzwa et al. [61] also showed that the use of ultrasound can significantly increase the vitamin C content in the vacuum-impregnated material without affecting other impregnation parameters. Interestingly, the researchers reported that when ultrasound (35 kHz, 240 W) was applied during the reduced pressure step, there was no statistically significant difference in vitamin C retention if compared to material treated with vacuum impregnation without ultrasonic treatment. However, in the case of ultrasonic treatment with the same parameters applied during the relaxation phase or during both stages of vacuum impregnation, a significant increase in the content of vitamin C was reported. The authors stated that the possible cause of this phenomenon is the occurrence of two types of cavitation – stable (without implosion of gas–vapor bubbles) and unstable (with bubble implosion) [61]. Moreover, the content of vitamin C was correlated (r = 0.85, p < 0.05) with the TPC value, while TPC was positively correlated (r = 0.74, p < 0.05) also with antioxidant activity.

Regarding the total polyphenols content (Fig. 4b) the application of US at the frequency of 25 kHz resulted in a further decrease of TPC in the impregnated samples. This is in contrast to a study conducted by Rahaman et al. [62], who observed an increase in the content of polyphenols as a result of ultrasonic treatment (25 kHz, 30 or 60 min) of plums in sucrose or glucose solutions. Similarly, Dinçer [40] observed that regardless of the vacuum pressure used (100, 250, 500 mbar), processing supported by ultrasound at a frequency of 40 kHz led to an increase in the anthocyanin content in apples impregnated with hibiscus juice [40]. In the present study, the TPC content in the US treatment at 45 kHz was at a similar level as in the vacuum impregnated samples. Different behaviour of samples subjected to two US frequencies could be caused by different changes in structure that could have increased the leakage of cell juice from the apple tissue into the impregnating solution or hindered the penetration of the impregnating solution into the pores.

The antioxidant activity of fresh apples was 3.68±0.20 mg Trolox/g d.m. and remained almost unchanged after application of vacuum impregnation in aloe juice. Expect for the samples treated by US at 45 kHz for short period, US generally caused a significant decrease in the antioxidant activity of the VI apple samples. As explained above for the bioactive compounds content the cause of this phenomenon may be the bidirectional mass transfer during the VI process [50], [63]. In the first stage of the VI process, there is a partial loss of native bioactive compounds from the porous tissue, while in the second stage of the process, external bioactive substances could penetrate the tissue together with the impregnating solution. Due to changes in the structure following the US treatments, the mass transfer may increase in both stages of VI and additionally, mass transfer also occurs during US treatment itself. Interesting results were presented by Yılmaz and Bilek [50], who vacuum impregnated apples in a solution containing 0.2 M mannitol, 3 % calcium lactate and 3 % lactic acid, with or without the addition of black carrot concentrate. The VI process was supported by ultrasound with a power of 96, 130 or 158 W. In the case of the solution containing black carrot concentrate, an increase in antioxidant activity was observed with the increase in ultrasound power. When the solution did not contain black carrot concentrate, the antioxidant activity of apples decreased with increasing ultrasound power [50]. In fact, the composition of the external impregnating solution may have a crucial role in the final composition of the samples in terms of bioactive compounds and final antioxidant activity.

### 3.5 Influence of ultrasound application and vacuum impregnation process on functional groups in the molecules (evaluated with FTIR) and thermal stability (TGA)

**Fig. 5:**
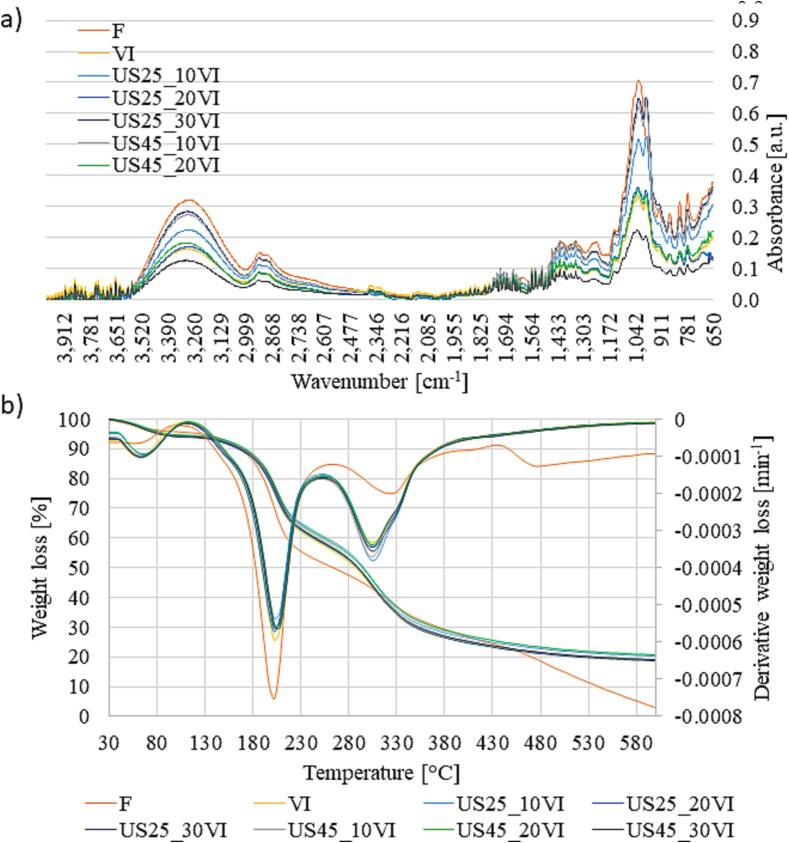
Influence of ultrasound application and vacuum impregnation process on a) functional groups in the molecules evaluated with FTIR; b) thermal stability evaluated with TGA.

Fig. 5a shows the FTIR spectra of untreated and vacuum impregnated samples with or without the application of ultrasound. The spectra shows the absorption bands at different wavelengths reflecting their functional groups [64]. All the tested samples showed a similar peak distribution, which means the presence of the same functional groups within the samples. This probably is due to the fact that the same raw material (apple) was used for each treatment and the aloe vera juice used for the impregnation did not introduce other functional group into the samples. Due to the different treatment applied a change in peaks heights were observed reflecting the changes in structure and chemical composition. The mid-IR spectra can be divided into four regions: the single bond region (2500–4000 cm^−1^), the triple bond region (2000–2500 cm^−1^), the double bond region (1500–2000 cm^−1^), and the fingerprint region (600–1500 cm^−1^) [65].

From the Fig. 5a it can be seen some small oscillations in the range of 4000–3600 cm^−1^ indicating the presence of water molecules in the tested samples. A wide band was observed in the range of 3600–3000 cm^−1^ a wide, corresponding to the presence of hydroxyl groups, which may originate from alcohols, phenols or carboxylic acids [66]. The absorbance band around 2950 and 2855 cm^−1^ indicates long-chain linear aliphatic compounds [65]. The small vibration in the range of 1750–1650 cm^−1^ can be attributed to the carbonyl group (C = O) that are characteristic of aldehydes, esters and aromatic acids. As reported by Kowalska et al. [67], the absorbance peaks in the range of 1450–1300 cm^−1^ can indicate the presence of alkanes. In addition, the bands that appeared in the range of 1270 cm^−1^ and 1200 cm^−1^ are attributed to the aromatic ethers and organic phosphates [64]. C–O or C–C stretching vibrations were found in the 900–1150 cm^−1^, indicating the presence of carbohydrates (polysaccharides cellulose) and other various groups of saccharides, such as glucose, fructose and sucrose [64].

All treatments applied resulted in a decrease in high of the most representative picks and bands, however the most similar behaviour to the fresh samples was observed in samples treated with US (25 kHz, 30 min; 45 kHz, 10 min) before vacuum impregnation. Wahid et al. [64] observed an increase in high of the spectra following the vacuum impregnation treatment of broccoli, however they used an impregnating solution with variable concentration of calcium chloride (CaCl_2_) and ascorbic acid.

Fig. 5b shows the weight losses of untreated and vacuum impregnated samples with or without the application of ultrasound, depending on the phase of thermal degradation. All samples showed four phases of thermal decomposition. Phase I corresponds to the loss of water and volatile substances and occurs between 30 and 110 °C. In our study the samples used for the TGA analysis were previously freeze-dried therefore the weight decrease was relatively low, around 4.5 % for untreated samples and 4.6–6.1 % for treated ones. Phase II (110–250 °C) corresponds to the degradation of mono- and disaccharides such as fructose (119–165 °C), glucose (150–200 °C) and sucrose (170–212 °C) [68]. During this phase a weight loss ranged from 33.3 to 43.5 % and it was the largest losses obtained in thermogravimetric analysis. The highest loss was observed in the untreated samples, while VI process decreased the degradation of sugars. The application of US before VI treatment resulted in even greater decrease in this parameter. Since the apple tissue mainly contains glucose and fructose, it can be assumed from the thermogravimetric analysis that ultrasound-assisted vacuum impregnation mainly led to a lower loss of monosaccharides from the tissue.

Phase III (250–440 °C) represents the degradation of polysaccharides such as hemicellulose 220–315 °C, cellulose 250–450 °C and lignin 320–450 °C [69], [70]. During this phase the mass losses amounted to about 28.4–36.8 %. The untreated apples showed the lowest losses (28.4 %), while the VI process increased losses to 35.1 %. A greater weight loss in phase III in the case of apples impregnated in aloe vera juice solution compared to untreaded apples may be due to the presence of other polysaccharides in the impregnating solution. In fact, the aloe vera juice contains, among others, the polysaccharides such as acemannan, which is mainly degraded at temperatures from 200 to 600 °C [71]. Pre-treatment with ultrasound resulted in an increase in mass loss in this phase ranging from 35.5 to 36.8 %. These results could be the indication of better impregnation of apples with aloe vera juice thanks to the action of ultrasound. Phase IV (440–600 °C) corresponds to the decomposition of substances formed by polymerisation of the degradation products generated in the earlier steps [68]. In this phase the mass losses were much higher in untreated samples (20.6 %) that in the vacuum impregnated ones (4.0–7.2 %).

### 3.6 Cluster analysis and principal components analysis

**Fig. 6:**
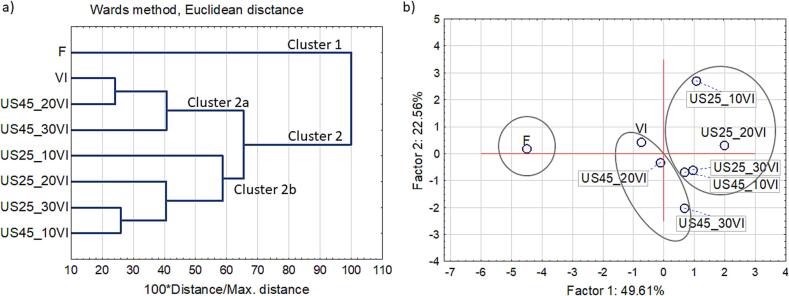
Influence of ultrasound application and vacuum impregnation process a) Cluster analysis; b) Principal Components Analysis (PCA).

The outcomes of the Cluster Analysis and Principal Components Analysis (PCA) are shown in Fig. 6. The analysis was carried out taking into account various factors such as dry matter content, water activity, color parameters (L*, ΔE, BI), hardness, total polyphenol content, vitamin C content, and antioxidant activity. The results of the analysis made it possible to distinguish between two large groups (clusters). Cluster 1 included fresh samples, while cluster 2 included samples subjected to VI, regardless of whether US pre-treatment was performed or not. This means that the VI process significantly affects the properties of the final product. All VI samples located in cluster 2 can be divided into two subgroups: cluster 2a, which includes vacuum-impregnated apples without additional treatments, and those treated with US treatment of 45 kHz for 20 and 30 min, and cluster 2b, which includes materials after vacuum impregnation using US treatment of 45 kHz for 10 min and all treatments with US of 25 kHz, regardless of the treatment duration. The results indicate that the type of US processing has a significant impact on the quality of vacuum impregnated products. The parameters of these treatments (frequency, time, intensity) lead to differences in the properties of the obtained materials. It should also be emphasized that, based on the included analyses, the samples prepared with pre-treatment with US at a frequency of 45 kHz and lasting 20 or 30 min were the least dissimilar to the vacuum-impregnated material.

## 4 Conclusions

The use of ultrasonic pre-treatment allowed to increase the effectiveness of vacuum impregnation process and to increase the percentage of weight gain and the dry matter content of apple slices, due to greater penetration of substances dissolved in the impregnating solution. As a result of vacuum impregnation and US treatment, a clear color change was observed compared to the fresh apple tissue, resulting in total color difference in the range of 28.93 – 34.17 and a decrease in L* parameter value and an increase in browning index. Regarding the bioactive compound content and antioxidant activity, generally, the effect of US treatment was strongly dependent on the applied process parameters. Vitamin C content was preserved when US was applied at 45 kHz for at least 20 min, while TPC decrease in all the treated samples, with values most similar to the fresh samples when US was applied at 45 kHz. In contrast, the antioxidant activity was unchanged in the VI samples and those treated with 45 kHz for short periods. These alterations in the chemical compositions of the material after US and VI processing was noticed also by TGA (Thermogravimetric Analysis) and FTIR (Fourier Transform Infrared) analyses. Reduced content of bioactive compounds can be linked to the structure changes after US processing, which, on the one hand, may support mass exchange and, on the other hand, cause the loss of nutritionally valuable substances. Thus, the US treatment applied before VI can be a suitable process, provided appropriate processing parameters are established for the intended purposes.

## CRediT authorship contribution statement

**Magdalena Trusinska:** Writing – original draft, Visualization, Validation, Software, Methodology, Investigation, Formal analysis, Data curation, Conceptualization. **Katarzyna Rybak:** Writing – original draft, Validation, Methodology, Investigation. **Federico Drudi:** Writing – original draft, Validation, Software, Methodology, Investigation. **Urszula Tylewicz:** Writing – review & editing, Writing – original draft, Supervision, Resources, Methodology, Funding acquisition, Conceptualization. **Malgorzata Nowacka:** Writing – review & editing, Writing – original draft, Supervision, Project administration, Methodology, Investigation, Funding acquisition, Conceptualization.

## Declaration of competing interest

The authors declare that they have no known competing financial interests or personal relationships that could have appeared to influence the work reported in this paper.
